# Neuronal calcium signaling pathways are associated with the development of epilepsy

**DOI:** 10.3892/mmr.2014.2756

**Published:** 2014-10-23

**Authors:** FANXIN MENG, YU YOU, ZHILIANG LIU, JIANMING LIU, HU DING, RUXIANG XU

**Affiliations:** Department of Neuronal Surgery, The Military General Hospital of Beijing PLA, Beijing 100007, P.R. China

**Keywords:** epilepsy, differentially expressed miRNAs, target gene, pathway, rat model

## Abstract

Epilepsy is the most common serious neurological disorder worldwide, however, the specific causative factors and mechanisms underlying epilepsy remain unclear. The current study aimed to study the potential genes or pathways associated with epilepsy, based on rat miRNA expression profiles. The microarray dataset GSE49850 was downloaded and analyzed with the TimeCourse R software package, which was used to generate comparisons between the control and electrically-stimulated groups. The target genes of differentially expressed miRNAs were queried in the miRWalk database and functional enrichment was conducted using the Database for Annotation, Visualization and Integrated Discovery software tools. The interaction network of the target genes was constructed based on the Biomolecular Interaction Network Database and clustered using ClusterONE. In total, 152 differentially expressed miRNAs were identified, with rno-miR-21-5p being the most significantly differentially expressed. A total of 526 target genes of the differentially expressed miRNAs were obtained. Functional analysis indicated that these genes were predominantly involved in responses to stimuli. The interaction network showed that the GRIN and STX gene family, which are involved in synaptic signal transmission, were significant. In conclusion, the present study identified that the development of epilepsy was closely associated with neuronal calcium signaling pathways.

## Introduction

Epilepsy is the most common serious neurological disorder worldwide (1). It is characterized by recurring and unprovoked seizures with a prevalence of 1% and a lifetime incidence of 3% (2). The high drug-resistance rate (3) in epilepsy creates a great financial burden for society, hence there has been much research devoted to the study of the mechanisms of epilepsy (4). Epilepsy is associated with brain neurons which are activated upon initiation of neuronal damage cascade reactions (5,6). There have been numerous advances in the treatment of epilepsy, including the generation of drug treatments (7), an increasing number of investigations (8) and the establishment of novel surgical approaches (9). The specific causative factors and detailed mechanisms of epilepsy remain unclear.

Micro RNAs (miRNA) are a group of non-coding RNAs (20–30 nucleotides in length) that regulate the expression of target genes by binding to the 3′-untranslated regions of target messenger (m)RNAs (10,11). The majority of mRNAs are regulated by miRNAs, and each miRNA can target hundreds of genes (12). A previous study indicated that 60% of all miRNA species are expressed in the brain (13). Thus, miRNAs may be involved in various biological processes associated with brain funciton, including learning, memory, neurological diseases (14) and neuroprotection (15).

A study on the metabolic pathways affected by epileptic processes demonstrated that miRNAs are particularly important in epigenetic mechanisms (16). A dysfunction of processing, variable expression levels and alterations in the expression of individual miRNAs were observed in the temporal lobe of patients with epilepsy together with hippocampal sclerosis (17). Bot *et al* (18) measured the miRNA levels in the dentate gyrus in epileptic rats and suggested that miRNAs can participate in several molecular events that occur in epileptic tissue, including the immune response and neuronal plasticity. It was hypothesized that complex changes in the expression levels of miRNAs suggest an important role for miRNAs in the molecular mechanisms of epilepsy. However, data regarding the role of miRNAs in epilepsy are limited.

In the current study, a bioinformatic-based analysis was performed on the miRNA expression profiles in rat models of epilepsy, with the aim to reveal the potential genes or pathways associated with epilepsy.

## Materials and methods

### miRNA array data

The expression profile under the accession number GSE49850 (18) was downloaded from the public functional genomics data repository, Gene Expression Omnibus, which was based on the GPL17566 platform (miRCURY LNA microRNA array; Exiqon, Woburn, MA, USA). A total of 686 miRNAs in rats were analyzed in GSE49850. Data sets consisted of miRNA expression levels at 7, 14, 30 and 90 days subsequent to electrical stimulation of the amygdala, which was used as a model of temporal lobe epilepsy. The sham-operated time-matched control group (N) and the stimulated group (C) were included at each time point with 5 replicates of each.

### Differentially expressed miRNA screening

The R statistical software package was used to analyze the gene expression profiles. The CEL source files were processed into expression estimates and a background correction was performed with the normexp method and quartile data normalization using the Robust Multiarray Average algorithm (19). The probability of genes being differentially expressed between human degenerative disc disease samples and normal samples was computed using the limma package (20). The TimeCourse software (21,22) was used to analyze the time-course microarray data and differences in four time points in the N and C groups. Hotelling’s T^2^ test was used to identify differentially expressed genes. P<0.01 was considered to indicate statistically significant data.

### Expression pattern analysis

The mfuzz package (23) in Bioconductor was used for miRNA cluster analyses in the C and N groups at four time points. Cluster analysis of the differential expression values in the 40 samples was performed with a Euclidean distance, using Cluster 3.0 software (24).

### Predication and enrichment of target genes

The names of differentially expressed miRNAs were matched to the miRBase (25) to identify the accession IDs. The target genes associated with miRNAs were then selected using the miRWalk database (26).

The enrichment of all target genes was conducted using the Database for Annotation, Visualization and Integrated Discovery (DAVID) software (27,28). This program has been widely used to investigate the bio-functions of genes based on the gene ontology (GO) and the Kyoto Encyclopaedia of Genes and Genomes (KEGG) databases. In the present study, DAVID was used to perform the GO and KEGG pathway enrichment analysis. The biological process, molecular function and cellular components were included in the enrichment analysis. In each category, there were at least 5 genes with P<0.01.

### Construction of target gene network

Genes and proteins associated with the target genes were screened in the BIND (29) database with the BisoGenet (30) package from the Cytoscape (31) software to obtain the interaction network. Additionally, ClusterONE (32) in Cytoscape was used for the cluster analysis. P<0.05 was considered to indicate statistically significant data.

## Results

### Differentially expressed miRNAs in the N and C groups

**Figure 1:**
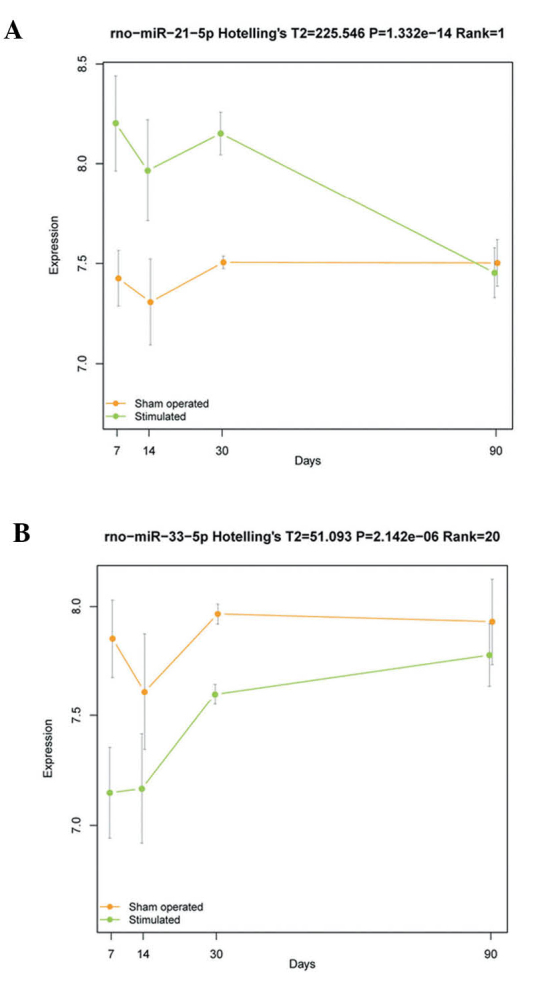
Expression of the most significant up- or downregulated miRNAs in each time point in the stimulus group. (A) Upregulated; and (B) downregulated miRNAs. Orange, sham operated; Green, stimulated.

The microarray data were processed with background correction and standardization to obtain the differentially expressed miRNAs. At a P-value of <0.01, a total of 152 differentially expressed miRNAs (including 144 up- and 8 downregulated) were obtained with Hotelling’s T^2^ test. The top 10 most significantly differentially expressed miRNAs are listed in Table I. The rno-miR-21-5p and rno-miR-33-5p were the most significantly up- and downregulated differentially expressed miRNAs, respectively (Fig. 1).

### Cluster analysis

**Figure 2:**
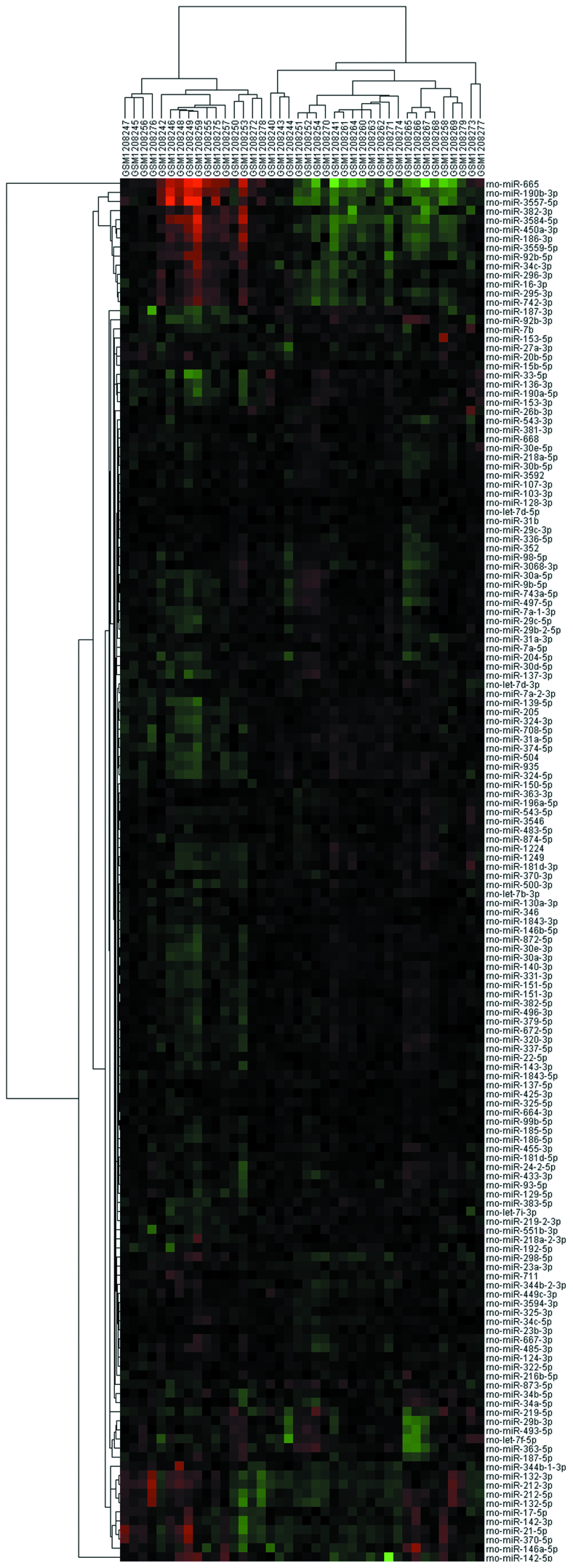
Expression clustering map of 152 differentially expressed microRNAs in all chip samples.

**Figure 3:**
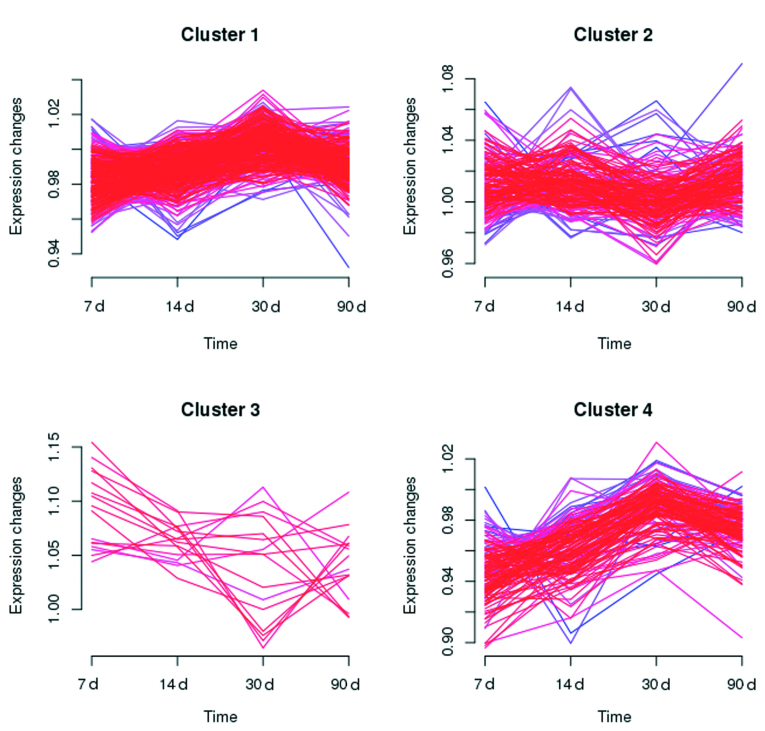
Top four microRNA expression clustering. Cluster 1 and 4 represent slow and rapid increases in the expression, respectively; Cluster 2 and 3 represent slow and rapid reductions in the expression, respectively.

The expression patterns of 152 differentially expressed miRNA in 40 samples exhibited a significant distribution character (Fig 2). Multiple clustering between the N and C groups was carried out with mfuzz at four time points (Fig. 3). Clusters 1 and 4 represent slow and rapid increases in the expression, respectively; whilst clusters 2 and 3 represent slow and rapid reductions in the expression, respectively.

### Target gene analysis

**Figure 4:**
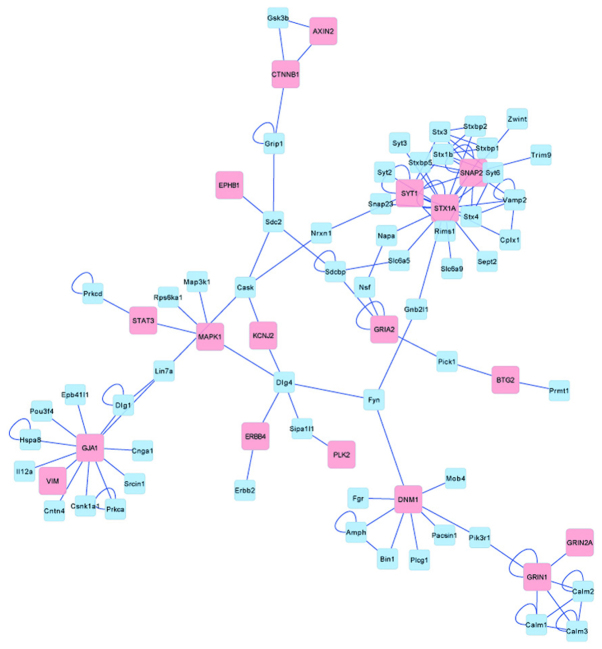
Interaction network for the target genes of the differentially expressed microRNAs. Pink, target genes; blue, genes associated with target genes.

**Figure 5:**
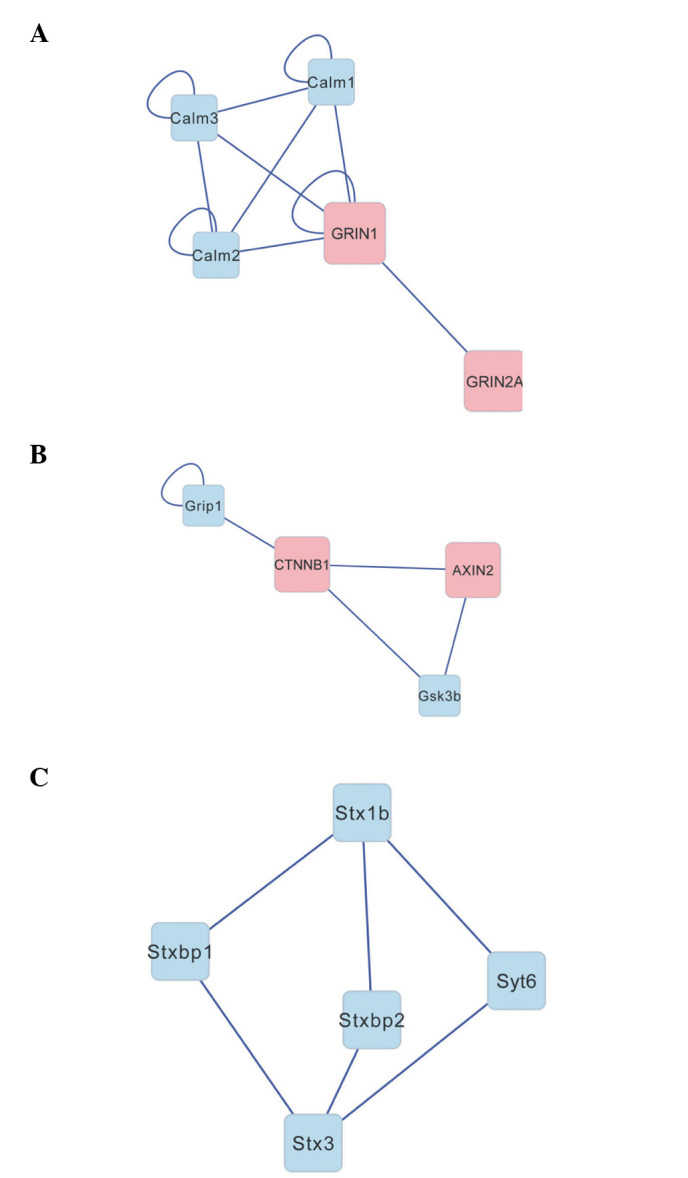
Three modules extracted from the interaction network of target genes in (A) cluster 1; (B) cluster 2; and (C) cluster 3; Pink, target genes; blue, genes associated with target genes.

Following removal of the duplicate genes, 526 target genes associated with miRNA were identified. The interaction network (Fig. 4) was constructed when these target genes were matched to the BIND database. The result of the network construction revealed that a total of 18 target genes, including GRIN1, GRIN2A, STX3, STX1A, STX1B, MAPK1 and Calm 1–3, were included in the network. The cluster analysis by ClusterONE software revealed three significant categories (Fig. 5), including: GRIN and Calm (density=0.7, quality=0.875, P=0.007); CTNNB1 and AXIN2 (density=0.667, quality=0.8, P=0.019); and the STX family (density=0.6, quality=0.429, P=0.049).

GO terms and KEGG pathway enrichment analysis indicated that there were 30 KEGG pathways and 630 GO terms in 113 types of cluster categories, including external stimuli response, gene transcription regulation and steroid response. The 3 most significantly enriched terms in each category are described in Table II.

## Discussion

Epilepsy is the most common serious neurological disorder worldwide (1), and the pathogenesis of the disease remains unclear. A bioinformatics analysis based on the expression profiles of miRNAs in epilepsy was performed to reveal the potential genes or pathways associated with epilepsy. The results of the present study demonstrated a total of 152 differentially expressed miRNAs, and 526 target genes of the differentially expressed miRNAs. Functional analysis indicated that these genes were largely involved in stimulus responses. The interaction network clarified that the GRIN and STX gene family, which are associated with synaptic signal transmission, had a significant interaction.

As important biological indicators, the expression of miRNAs has been proved to be strongly associated with various diseases, including brain tumors (33), breast cancer (34), lung tumor (35) and Alzheimer’s disease (36). In studies of epilepsy, the association between the expression of miRNAs and the processes of the disease have been demonstrated in humans (17) and animal models (18). In previous pilocarpine-induced status epilepticus studies in rat models, authors have theorized that the majority of miRNAs were downregulated following pilocarpine application (37), while other studies indicated that the majority of miRNAs were upregulated (38). In the present study, the majority of the 152 differentially expressed miRNAs were upregulated, including miR-21. miRNA-21 is able to mediate tumor growth (39). A number of previous studies have indicated that the downregulation of miRNA-21 is strongly associated with the processes of tumors or cancer (40–42). However, miRNA-21 was significantly upregulated in the present study as a differentially expressed miRNA. This result indicates that the upregulation of miRNA-21 may be involved in the initiation of the cell signaling pathways associated with epilepsy, which is supported by a study by Marquez *et al* (43). Furthermore, the expression of target genes associated with these differentially expressed miRNAs were mostly downregulated. Bot *et al* (18) indicated that the target genes may be involved in several biological functions, including regulation of transcription, wound response, apoptosis, cell proliferation and immune response. The enrichment analysis of target genes in the present study demonstrated that their function is to respond to stimuli such as macromolecular substances and hormones. With the downregulation of target genes, the external stimuli response for cells is weakened. Thus, the signal response pathways in these cells may be damaged. This may be one reason for the neuronal dysregulation observed in the epileptic brain (44).

In the present study, the cluster analysis of the target genes in the network construction revealed three significant categories including the GRIN, Calm and STX families. N-methyl-D-aspartate (NMDA) receptors are vital for healthy brain development (45). The NMDA-type glutamate receptors GRIN1 and GRIN2A, are two members of the GRIN family. Mutations in GRIN2A can induce idiopathic focal epilepsy (46), while GRIN1 has been associated with schizophrenia susceptibility (47). Calm1–3 are three members of the calmodulin family (48) that are associated with calcium signaling receptor activation. The sialyltransferase family (STX) (49) consists of various members such as STX3, STX1A and STX1B. These influencial factors, in particular STX1A, are involved in the regulation of synaptic vesicles, which are used to store neurotransmitters. As a target gene for differentially expressed miRNA, STX1A is a key regulator of ion channels of the nervous system, although it was not included in the cluster categories in the present study.

The results of the interaction network for these target genes indicated that signal transduction of normal nerve cells suffers significant interference in the process of epilepsy, thus the nerve cells may not function properly, leading to the occurrence of disease. Understanding the mechanisms of the differential expression of miRNAs in epilepsy is crucial for understanding the pathophysiology of epilepsy and may constitute interesting candidate targets for therapy. However, to understand the mechanism of the different miRNAs on the function of epilepsy requires in-depth knowledge of the targets of each miRNA, and a larger scale of miRNA-target gene screening. In the current study, the targets of the miRNAs were unknown and require further investigation.

In conclusion, the present study, based on miRNA expression profiles, demonstrated that the development of epilepsy is closely associated with the neuronal calcium signaling pathways involving the GRIN and STX families. The results suggested an important function for miRNAs in molecular mechanisms of epilepsy, and provided a potential direction for future research into the disease, and its treatments.

## Figures and Tables

**Table I:** Top 10 most significantly differentially expressed microRNAs.

microRNA	Hotelling’s T^2^	P-value
rno-miR-21-5p	225.54606	1.332268 × 10^−14^
rno-miR-218a-5p	215.12361	2.697842 × 10^−14^
rno-miR-370-5p	176.86949	4.736211 × 10^−13^
rno-miR-132-5p	133.86876	2.341205 × 10^−11^
rno-miR-212-3p	101.48246	8.938348 × 10^−10^
rno-miR-212-5p	96.00433	1.796583 × 10^−9^
rno-miR-298-5p	89.53393	4.253404 × 10^−9^
rno-miR-352	85.14339	7.825937 × 10^−9^
rno-miR-363-5p	80.30582	1.571388 × 10^−8^
rno-miR-124-3p	74.28944	3.892660 × 10^−8^

P<0.01 was considered to indicate statistical significance.

**Table II:** Top 3 most significant Gene Onology terms and KEGG pathway enrichment.

Category	Term	Count	Fold enrichment	FDR
Biological process	GO:0010033~response to organic substance	135	4.238731	1.45E-47
	GO:0009719~response to endogenous stimulus	93	4.729099	2.14E-34
	GO:0009725~response to hormone stimulus	85	4.856225	7.51E-32
Cellular component	GO:0031974~membrane-enclosed lumen	96	2.246048	1.55E-11
	GO:0043233~organelle lumen	93	2.236448	6.30E-11
	GO:0044421~extracellular region part	64	2.711679	4.82E-10
Molecular function	GO:0046983~protein dimerization activity	69	3.918162	1.51E-19
	GO:0003700~transcription factor activity	73	3.398632	2.97E-17
	GO:0019899~enzyme binding	57	3.473577	5.11E-13
KEGG pathway	rno05210:Colorectal cancer	23	6.0353	8.78E-09
	rno05220:Chronic myeloid leukemia	20	5.667934	9.28E-07
	rno05215:Prostate cancer	20	4.723278	2.46E-05

P<0.01 was considered to indicate statistical significance. FDR, false discovery rate; fold enrichment, proportion of significant genes in the specified term vs. overall proportion of significant genes; KEGG, Kyoto Encyclopaedia of Genes and Genomes.
